# Sunlight active formulated extract (SAFE): an Egyptian invention for malaria vector control

**DOI:** 10.1186/1475-2875-11-S1-P43

**Published:** 2012-10-15

**Authors:** Tarek A El-Tayeb, Mahmoud H Abdel-Kader

**Affiliations:** 1 Faculty of Pharmacy and Biotechnology, German University in Cairo, New Cairo, Egypt; 2 National institute of Laser Enhanced Sciences (NILES), Cairo University, Cairo Egypt

## 

This invention introduces an innovative modality for malaria vector control that combines both effectiveness and efficiency with the highest levels of human safety and environment friendliness.

The Novality of this (PCT) patent lies in developing a new ecologically safe modality using a natural plant extract (chlorophyll derivatives) and mosquito larvae attractant as sunlight active photo-larvicide for outdoors control of the *larvae of Anopheles gambiae*, *Culex pipiens and Aedes aegypti.* In this context, the accumulated photoactive compound (photosensitizer) in the larvae body induces, upon sunlight exposure, an oxidation stress that results in organism death. The obtained results reveal that SAFE is highly effective as it ensures up to 100% mortality of mosquitoes larvae. The effectiveness of SAFE accumulation in the larvae bodies was qualitatively and quantitatively investigated using the Confocal Laser Scanning Microscopy (CLSM) technique. The active ingredient of SAFE (chlorophyll derivative) exhibit several advantages: they are low cost, natural products extracted from green plants and endorsed by the Food and Drug Administration (FDA) as food additives. In addition, they are used in very low concentrations (µM), can be easily applied in the field by being dissolved in an aquatic environment and most importantly they are highly effective.

